# 0988. Human neutrophil peptides play an important role in the pathogenesis of ARDS

**DOI:** 10.1186/2197-425X-2-S1-P73

**Published:** 2014-09-26

**Authors:** F Pozzi, M Parotto, R Molinaro, D Islam, A Pesenti, AS Slutsky, H Zhang

**Affiliations:** Keenan Research Centre for Biomedical Science of St. Michael's Hospital, Toronto, Canada; University of Milan - Bicocca, Milan, Italy

## Introduction

Acute respiratory distress syndrome (ARDS) and ventilator induced lung injury (VILI) are characterized by neutrophil recruitment in the lung, and human neutrophil peptides (HNP) are the most abundant cationic proteins that are released into the extracellular matrix upon neutrophil activation. We and others have previously shown that high concentrations of HNP can lead to capillary-epithelial barrier damage in a mouse model of ARDS. We also demonstrated that VILI can lead to lung fibrotic development. We now extended the studies to examine the effects of HNP in lung remodeling.

## Objective

Examine the role of HNP on lung remodeling in a mouse model of ARDS followed by mechanical ventilation.

## Methods

Mice endogenously expressing HNP (HNP^+/+^) were generated using a human neutrophil elastase promoter. The HNP^+/+^and the FVB wild type mice received either HCl (pH = 1.0, 3 mL/kg) intratracheally, or vehicle solution as a control. Respiratory system compliance, protein concentrations and differential cell counting in bronchoalveolar lavage fluid (BALF) were determined 48 h after HCl. In a parallel experiment, the mice were randomized into 3 different groups 48 h after HCl: 1) no mechanical ventilation; 2) low pressure mechanical ventilation for 2 h; and 3) high pressure mechanical ventilation for 2 h. The mice were then observed for 14 days for evaluation of epithelial damage, Ashcroft score for fibrosis and inflammation.

## Results

**Figure 1:**
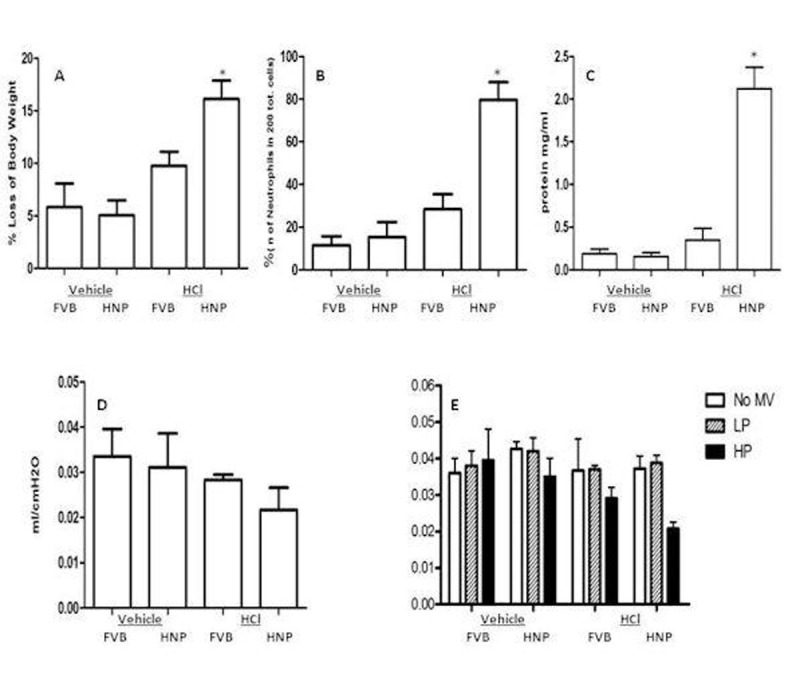
HNP contributes to lung injury. A. Loss of body weight. B. higher neutrophil count. C. increased total protein concentration in BALF, and D. decreased lung compliance in HNP^+/+^ mice 48h after HCI (3 ml/kg) challenge. E. Lung lung compliance remained low in HNP^+/+^ mice 14 days ater HCI installation and mechanical ventilsation (MV) at either low pressure (LP) or high pressure (HP). *p<0.05 as compared to other groups.

The HNP^+/+^ mice showed significantly body weight loss (Fig 1A), increased neutrophil count (Fig 1B) and enhanced total protein (Fig 1C) in BALF and decreased lung compliance (Fig 1D) at 48 h after HCl challenge, as compared to the FVB mice. The lung compliance remained low in HNP^+/+^ mice 14 days after HCl instillation followed by mechanical ventilation at either low pressure or high pressure (Fig 1E).

## Conclusion

HNP can accelerate inflammation at an early phase in a mouse model of HCl-induced ARDS, which may contribute to lung remodeling at late phases, magnifying the deleterious effects of ventilator-induced lung injury. We are hoping to present a full picture regarding lung remodeling 14 days after receiving HCl/mechanical ventilation at the ESICM conference in September.

